# Ictal Epileptic Headache: When Terminology Is Not a Moot Question

**DOI:** 10.3389/fneur.2019.00785

**Published:** 2019-07-23

**Authors:** Pasquale Parisi, Maria Chiara Paolino, Umberto Raucci, Nicoletta Della Vecchia, Vincenzo Belcastro, Maria Pia Villa, Pasquale Striano

**Affiliations:** ^1^Chair of Pediatrics, Child Neurology, NESMOS Department, Faculty of Medicine and Psychology, Sapienza University, Sant'Andrea Hospital, Rome, Italy; ^2^Pediatric Emergency Department, Bambino Gesù Children's Hospital, IRCCS, Rome, Italy; ^3^Department of Pediatrics, University of Campania Luigi Vanvitelli, Naples, Italy; ^4^Neurology Unit, Department of Neuroscience, Sant'Anna Hospital, Como, Italy; ^5^Department of Neurosciences, Rehabilitation, Ophthalmology, Genetics, Maternal and Child Health, University of Genoa, Genova, Italy; ^6^Paediatric Neurology and Muscular Diseases Unit, G. Gaslini' Institute, Genova, Italy

**Keywords:** ictal epileptic headache, hemicrania epileptica, epilepsy, migraine, tension-type headache, EEG, autonomic seizures, panayiotopoulos syndrome

## Abstract

The relationship between headache and epilepsy is complex and despite the nature of this association is not yet clear. In the last few years, it has been progressively introduced the concept of the “ictal epileptic headache” that was included in the recently revised International Classification of Headaches Disorders 3rd edition (ICHD-3-revised). The diagnostic criteria for ictal epileptic headache (IEH) suggested in 2012 were quite restrictive thus leading to the underestimation of this phenomenon. However, these criteria have not yet been included into the ICHD-3 revision published in 2018, thus creating confusion among both, physicians and experts in this field. Here, we highlight the importance to strictly apply the original IEH criteria explaining the reasons through the analysis of the clinical, historical, epidemiological and pathophysiological characteristics of the IEH itself. In addition, we discuss the issues related to the neurophysiopathological link between headache and epilepsy as well as to the classification of these epileptic events as “autonomic seizure.”

## Headache, Epilepsy, and “Ictal Epileptic Headache”

Headache and epilepsy are characterized by transient attacks of altered brain function. The links between headache and epilepsy are complex and in the last century there have been several attempts to improve the classification, the clinical characterization, and the physiopathology of this association (1, 2).

In the 20th century, Sir W. R. Gowers, first suggested that “migraine is in the borderland of epilepsy” (2), sharing some pathophysiological mechanisms, presenting themselves as dysfunctions of neurotransmitters and ion channels. Indeed, these two conditions are common, often co-morbid, with headache attacks in epilepsy, temporally related as pre-ictal, ictal, post-ictal, or inter-ictal events. In addition, they can present with either visual, cognitive, sensorial and motor signs/symptoms (1, 2). Furthermore, the concept of “headache” as “an epileptic headache” that “… may even be the only clinical manifestation of idiopathic epilepsy” dates back from a long time ago. Already in the pre-EEG era, Gowers stated that “…in extremely rare instances one affection may develop while the other goes on,” (2). Nowadays, the availability of digital EEG recordings allow us to state that chronic headache itself may occasionally represent an epilepsy condition and that often headache can represent the only ictal epileptic phenomenon (i.e., ictal epileptic headache, IEH) (3–6).

The first description of IEH dates to the 1950s (7–10) but the term migralepsy was coined 10 years later by Lennox and Lennox (11) and become strongly rooted in the epileptological culture, hindering the verification and awareness among experts of the possible complete overlap of an epileptic seizure and a cephalalgic event. In fact, following the introduction of the migralepsy concept, an increasing number of ictal headaches have been described (12–15) and it has been hypothesized that the migralepsy sequence may not exist at all and that the initial part of the “migralepsy” may merely be an “ictal headache” followed by other ictal autonomic, sensory, motor or psychic signs/symptoms, being thus classified in fact as “hemicrania epileptica” (16).

Although the revised International Classification of Headaches Disorders 3rd edition (ICHD-3) now includes the term “ictal epileptic headache” (17), it does not take into account the original clinical criteria (3). Indeed, since the first demonstration (13) of the immediate remission of EEG abnormalities and of the headache after the administration of intravenous anti-epileptic drug, over than 30 cases of IEH have been reported (3–6, 12–16, 18–34). These papers suggest that a diagnosis of IEH is possible when all the following criteria occur (3): (a) headache lasting minutes, hours, or days, headache that is ipsilateral or contralateral (if the headache is not generalized) with epileptiform EEG discharges (if the anomalies are localized); (b) variable EEG abnormalities may be observed without a specific EEG or clinical headache pattern required; (c) headache and EEG abnormalities immediately resolved after antiepileptic drugs intravenous administration (Table 1).

**Table 1:** Proposed original criteria for Ictal Epileptic Headache (IEH) [reproduce from Parisi et al. (3), with permission].

**Diagnostic criteria A–D must all be fulfilled in IEH**
A. Headache^*^ lasting minutes, hours, or days B. Headache that is ipsilateral or contralateral to lateralized ictal epileptiform EEG discharges (if EEG discharges are lateralized) B. Evidence of epileptiform (focal ^**^, lateralized or generalized) discharges on scalp EEG concomitantly with headache; different types of EEG anomalies may be observed (generalized spike-and-wave or polyspike-and-wave, focal or generalized rhythmic activity or focal sub-continuous spikes or theta activity that may be intermingled with sharp waves) with or without photoparoxysmal responses (PPRs) D. Headache and EEG abnormalities resolves immediately (within few minutes) after i.v. antiepileptic drugs administration

* *A specific headache pattern is not required (Migraine With or Without Aura, or Tension-type headache are all accepted)*.

** *Any localization (frontal, temporal, parietal, occipital) is accepted*.

However, these criteria have not been fully taken into account into the recent revised ICHD-3 (17), thus creating confusion. However, we feel that even if the concomitant appearance of the EEG epileptiform discharges with headache is the mainstay criteria for the diagnosis of IEH, the prompt response to antiepileptic treatment is still crucial to confirm the clinical suspicion (34).

Coci and Riedel recently described two patients, with chronic headache unresponsive to analgesics therapy and disappeared after oral antiepileptic therapy (24). An ictal EEG recording in these adolescents during a headache attack revealed diffuse spike-wave and poly-spikes, and spontaneous drug withdrawal resulted in a recurrence of the headache, which resolved again on anticonvulsant therapy. These authors classified these cases as “probable IEH” due to the lack of clinical-EEG demonstration of the resolution of both the headache and EEG anomalies, after the administration of intravenous anticonvulsant therapy. Therefore, although an EEG recording may not be routinely recommended in children with headache, it should be performed promptly in patients with prolonged headache that do not respond to anti-migraine therapy (35–39), particularly in children with epilepsy that also express other types of seizures (34). Nevertheless, the use of clear-cut IEH criteria (3) will facilitate communication among clinicians and researchers, avoiding misdiagnoses, incorrect therapies, and eventually reducing health costs (40–52).

## Cortical Dysexcitability and Cortical Spreading Depression: the Genetic and Neurophysiopathological Links Between Headache and Epilepsy

Several data support the view that increased neocortical excitability is the leading mechanism underlying headache and epilepsy (53). Taking into account that in migraine, during the “spreading depression,” hypo- and hyper-excitation occur, both (sequentially), as rebound phenomena, it could be suggested the term “dys-excitability” to better describe these physiopathologic events, rather than generically “hyperexcitability” (5, 6, 50, 54–57). Cortical Spreading Depression (CSD), that many Authors believe to be the most likely pathophysiological link between headache and epilepsy (5, 6, 36, 39, 40, 58, 59), is a slowly propagating wave of strong neuronal depolarization which induces fleeting (but intense) spike activity, followed by a neural suppression, lasting for minutes. The depolarization proceeds simultaneously with an increased regional cerebral blood flow, while the phase of reduced neural activity is associated with a decrease in brain perfusion. CSD starts the trigeminovascular system, provoking the release of many inflammatory molecules and neurotransmitters, responsible for the pain characterizing the headache phase (50, 60). Both, basic and clinical neurosciences findings, are in favor of “CSD” and “epileptic focus” as phenomenons able to facilitate reciprocally each other, although with different effectiveness and efficiency. The achievement of a minimum threshold necessary to start depolarization is the key to both phenomenas, but, the required threshold is presumed to be lower for CSD than for an epileptic discharge, the onset of both facilitating each other, anyway. This may explain why it is far more likely to observe an epileptic subject who also presents a peri-ictal headache than a cephalalgic patient who presents epilepsy (36, 40, 43, 50, 53, 57, 60). The two phenomena (CSD and epileptic seizure) possibly being triggered by more than one pathway converging upon the same destination: depolarization/dysexcitability (36, 40, 48, 50, 61).

The etiology could be environmental or individual (due genetic causes or not), originating a flow of ions that provokes CSD through neuronal and glial cytoplasmic bridges, rather than through interstitial ways as conversely occurs in the spreading of epileptic seizures (5, 50, 55, 56, 62).

Both migraine and epilepsy have an important genetic component, with strong evidence pointing to a shared genetic basis between headache and epilepsy emerging from clinical/EEG and genetic studies on Familial Hemiplegic Migraine (FHM) (63–69). Recent data suggest shared genetic substrates and phenotypic-genotypic correlations with mutations in some ion transporter genes, including CACNA1A, ATP1A2, and SCN1A (69–73). Other genetic findings pointing to a link between migraine and epilepsy have been published (74, 75). In addition, glutamate metabolism (76), serotonin metabolism (77), dopamine metabolism (78), and ion channel (sodium, potassium, and chloride) function might be impaired in both epilepsy and migraine (69, 79).

## In Most Cases IEH is Probably an “Autonomic Seizure”

To clarify why headache could be the sole ictal epileptic manifestation, we (3, 5, 6, 34) previously hypothesized that an autonomic seizure remains purely autonomic if ictal neuronal activation of non-autonomic cortical areas does not achieve the symptomatogenic threshold (80). Accordingly, we suggested that IEH should be considered an autonomic form of epilepsy, like Panayiotopoulos syndrome, and, thus, people with long-lasting IEH attacks may even fulfill the criteria for autonomic status epilepticus (81). Although it is difficult to explain the reasons for which IEH remains an isolated manifestation lasting up to several hours or even days (13), one can speculate that the threshold for ictal autonomic manifestations could be lower from that required for motor-sensory areas, as observed for autonomic seizures in pediatric age (e.g., Panayiotopoulos syndrome).

In addition, while the presence of epileptiform abnormalities usually confirm the diagnosis of epilepsy, in IEH patients the lack of clear epileptic spike-and-wave activity does not rule out the diagnosis of epilepsy. The same diagnostic difficulties arise for patients with a deep epileptic focus arising, for example, from the orbito-mesial frontal zone (82). In such cases, ictal epileptic EEG activity may be recorded exclusively by means of deep stereo-EEG recording, even, sometimes, purely by chance (83).

Another crucial point is the lack of a clear, repetitive EEG headache-associated pattern, owing to the fact that the ictal EEG recording is usually not associated with specific EEG picture. Indeed, different EEG patterns have been recorded during headache-like complaints in both symptomatic and idiopathic IEH cases (18, 20, 28–34).

Moreover, when EEG abnormalities are recorded, no specific cortical correlations emerge (e.g., focal frontal, parietal, temporal, occipital and primary or secondary generalized), as reported (confirming, thus, our hypothesis) for autonomic manifestations in Panayiotopoulos syndrome.

Accordingly, we may interpret a headache as the sole expression of an epileptic seizure, supporting thus the autonomic nature of the IEH, at least in the most of the cases.

## Further Neurophysiopathological Reflections on the Possible Link Between Autonomic and Headache Pathways

To understand the complexity of the pathways and networks involved in the onset and transmission of “primary headache” from the periphery (intracranial vessels) within the central nervous system until all potentially involved brain areas, you have to sum up the main stages of such nociceptive structures, fibers, pathways and such neuro-vascular structures. This careful examination can make evident why is so difficult, at moment, to classify the “Ictal Epileptic Headache” as “sensory” or “autonomic” seizure to propose a precise classification in the new Epilepsy classifications (84).

The cephalalgic attack originates as consequence of the activation of nociceptors innervating pial, arachnoid, and dural blood vessels, as well as large cortical arteries and sinuses. These structures are activated by mechanical, electrical or chemical stimulation (pro-inflammatory molecules, blood or infection), causing a painful perception similar to migraine and its most commonly associated symptoms/signs (nausea, throbbing pain, photophobia, and phonophobia).

The intracranial vessels and the meninges are innervated by unmyelinated fibers (C fibers) or thin little myelinated fibers (Ad fiber), which convey nociceptive sensitivity; these axonal terminations contain vasoactive neuropeptides such as substance P (SP) and the peptide related to the calcitonin gene (CGRP). They, originating from the trigeminal ganglion, reach the dura through the ophthalmic branch of the trigeminal nerve (V1) and, to a lesser extent, through the maxillary (V2) and mandibular branches (V3).

The dura is also innervated by neurons located in the ganglia of the upper cervical dorsal root. For decades, a possible vascular origin of headache pain has been debated. At present, the results of the various studies are conflicting and inconclusive, suggesting that vascular changes would not have a primary role, or at least, may not have a unique and predominant role in the pathophysiology of headache (85–87).

The mechanisms that explain the efficacy of the Vagus Nerve Stimulation (VNS) in the treatment of migraine and cluster headaches are not yet clear; probably, it is realized through a modulation of the intracranial trigeminal-vascular nociceptive transmission. Most of the fibers of the vagus nerve includes sensory afferents that terminate bilaterally in the nucleus tractus solitarius (NTS), before projecting into other nuclei, including the locus coeruleus (LC), the nucleus of the dorsal raphe (DRN), parabrachial nucleus, and PVN. It has been shown for the first time that VNS inhibits nociceptive activation of trigeminal-cervical neurons in preclinical models of acute dural-intracranial (migraine-like) and trigeminal-autonomic (cluster) pain (87).

The insula and other part of so-called Limbic System (part of frontal, temporal, and parietal regions which receive projections from autonomic networks), have a role in various processes including goal-directed cognition, conscious awareness, autonomic regulation, enteroception, and somatosensation. There are complex behaviors in migraine (conscious awareness and error detection), which are less investigated of other well-known, such as autonomic and somatosensory alterations during the clinical attacks. The insula processes and relays afferent inputs from brain areas involved in these functions, to areas involved in higher cortical function, such as frontal, temporal, and parietal regions. Insula role could be to decode the signals of altered internal milieu in migraine (along with other chronic pain conditions), taking into account the insula role in translating and integrating of multiple informations into complex behaviors (88).

It is also important to remember that, the activation of lateral and ventrolateral periacqueduttal gray (PAG) neurons by direct ascending lamina I e II projections (where make connection the afferent fibers C amyelinic which comes from cerebral vessels, as proposed for trigemino-vascular theory to explain physiopathology of migraine), produces non-selective, non-specific headache pain relief, cardiovascular reactions (decrease in blood pressure), homeostatic reactions (temperature changes), and defensive reactions (immobility, arousal, avoidance behavior, and vocalization), as well as a more general emotional state of fear and anxiety (89). Since the PAG undoubtedly projects a more dense fiber connections to the rostral ventromedial medulla (RVM), but minimally to the spinal and medullary dorsal horn, RVM neurons constitute a direct link for descending modulation through bilateral projections to all levels of spinal and medullary dorsal horns. These functional and anatomical studies are consistent with a broader modulatory role of the PAG–RVM circuit and suggest an “*absence of specificity*” for headache.

## Conclusions

IEH does not have a specific clinical picture of headache/migraine (migraine without aura or tension-type headache or aspecific headache patterns, have all been reported), and it can last from seconds to days, with evidence of synchronous ictal epileptiform EEG anomalies; different EEG patterns may be observed, with or without a photoparoxysmal response (see Table 1). In fact, in particular, the ictal EEG recording in most patients does not yield a particular EEG pattern or specific cortical topographic correlations (focal frontal, parietal, temporal, occipital, and focal with primary or secondary generalization, have all been reported). EEG recording is not recommended routinely in children with headache but should be considered promptly in case of prolonged migraine/headache not responsive to antimigraine drugs. If the main IEH criterion (EEG-clinical response to antiepileptic intravenous administration) is not satisfied, we can just pose a “probable IEH” diagnosis. The concept of migralepsy is potentially confusing and should not be used to describe the sequence of visual aura-seizure and an ictal EEG recording is mandatory in these patients to exclude an “hemicrania epileptica” (16).

## Author Contributions

PP, MP, and PS formulated original idea and the design of the review and wrote the first draft of the manuscript. ND, UR, VB, and MV approved the design and final version of the manuscript. All authors reviewed, approved, and agreed to be accountable for all aspects of the work.

### Conflict of Interest Statement

The authors declare that the research was conducted in the absence of any commercial or financial relationships that could be construed as a potential conflict of interest.
